# Transient neuroinflammation following surgery contributes to long-lasting cognitive decline in elderly rats via dysfunction of synaptic NMDA receptor

**DOI:** 10.1186/s12974-022-02528-5

**Published:** 2022-07-13

**Authors:** Bo Chen, Guangcheng Qin, Jingyu Xiao, Xiaoyuan Deng, Aolei Lin, Hongliang Liu

**Affiliations:** 1 Department of Anesthesiology, Chongqing University Cancer Hospital, Chongqing, 400030 People’s Republic of China; 2 Laboratory Research Center, the First Affiliated Hospital of Chongqing Medical University, Chongqing, 400016 People’s Republic of China; 3 Department of Neurology, the First Affiliated Hospital of Chongqing Medical University, Chongqing, 400016 People’s Republic of China

**Keywords:** Postoperative cognitive dysfunction, Neuroinflammation, NMDAR, structural plasticity, Rac1

## Abstract

**Background:**

Perioperative neurocognitive disorders (PNDs) are considered the most common postoperative complication in geriatric patients. However, its pathogenesis is not fully understood. Surgery-triggered neuroinflammation is a major contributor to the development of PNDs. Neuroinflammation can influence N-methyl-D-aspartate receptor (NMDAR) expression or function which is closely associated with cognition. We, therefore, hypothesized that the persistent changes in NMDAR expression or function induced by transient neuroinflammation after surgery were involved in the development of PNDs.

**Methods:**

Eighteen-month-old male Sprague–Dawley rats were subjected to abdominal surgery with sevoflurane anesthesia to establish the PNDs animal model. Then, we determined the transient neuroinflammation by detecting the protein levels of proinflammatory cytokines and microglia activation using ELISA, western blot, immunohistochemistry, and microglial morphological analysis from postoperative days 1–20. Persistent changes in NMDAR expression were determined by detecting the protein levels of NMDAR subunits from postoperative days 1–59. Subsequently, the dysfunction of synaptic NMDAR was evaluated by detecting the structural plasticity of dendritic spine using Golgi staining. Pull-down assay and western blot were used to detect the protein levels of Rac1-GTP, phosphor-cofilin, and Arp3, which contribute to the regulation of the structural plasticity of dendritic spine. Finally, glycyrrhizin, an anti-inflammatory agent, was administered to further explore the role of synaptic NMDAR dysfunction induced by transient neuroinflammation in the neuropathogenesis of PNDs.

**Results:**

We showed that transient neuroinflammation induced by surgery caused sustained downregulation of synaptic NR2A and NR2B subunits in the dorsal hippocampus and led to a selective long-term spatial memory deficit. Meanwhile, the detrimental effect of neuroinflammation on the function of synaptic NMDARs was shown by the impaired structural plasticity of dendritic spines and decreased activity of the Rac1 signaling pathways during learning. Furthermore, anti-inflammatory treatment reversed the downregulation and hypofunction of synaptic NR2A and NR2B and subsequently rescued the long-term spatial memory deficit.

**Conclusions:**

Our results identify sustained synaptic NR2A and NR2B downregulation and hypofunction induced by transient neuroinflammation following surgery as important contributors to the development of PNDs in elderly rats.

**Supplementary Information:**

The online version contains supplementary material available at 10.1186/s12974-022-02528-5.

## Introduction

Perioperative neurocognitive disorders (PNDs), encompassing acute delirium and long-lasting cognitive decline, are the most common postoperative complications that occur mainly in geriatric patients (> 65 years) [1–3]. PNDs are associated with extended hospital stay, a reduced quality of life, increased social transfer costs, and even higher mortality rates [1, 4, 5]. However, its pathogenesis is not fully understood. Accumulating evidence suggests a major contributor of surgery-triggered neuroinflammation to the development of PNDs, because transient changes in proinflammatory signaling molecules have been identified in both patients and animal models of PNDs [6–10]. Indeed, surgical trauma initially triggers the rapid release of damage-associated molecular pattern molecules, particularly high-mobility group box 1 (HMGB1) [9, 11], which then promote peripheral proinflammatory cytokine release from activated immune cells [12]. Increased peripheral proinflammatory cytokine levels subsequently disrupt blood–brain barrier (BBB) integrity, ultimately inducing neuroinflammation via cytokine expression and microglial activation [13–15]. High levels of proinflammatory signaling molecules can produce direct detrimental effects on cognitive function by attenuating long-term potentiation (LTP) [16], and this effect can be reversed by cytokine receptor antagonists [17]. In addition, transient neuroinflammation can lead to persistent changes in neuronal networks which are closely associated with cognition [16, 18, 19]. However, the exact molecular mechanism by which transient neuroinflammation after surgery causes PNDs, especially long-lasting cognitive decline, is not uncertain.

N-methyl-D-aspartate receptors (NMDARs) are glutamate-gated ion channels that are widely expressed in pyramidal neurons of the hippocampus [20] and play a vital role in excitatory synaptic transmission and plasticity, the underlying molecular mechanism of learning and memory [21–23]. NMDARs form functional on heteromeric complexes composed of obligatory NR1 and modulatory NR2 subunits (A–D) [24]. In addition, different subcellular localizations (synaptic or extrasynaptic) of NMDARs determine receptor properties and functions [25]. Changes in NMDAR expression or function are implicated in cognitive deficits resulting from a wide range of neuropathological disorders [26–28]. However, studies examining the regulatory effect of neuroinflammation on NMDARs have reported inconsistent results. For example, upregulation of NMDARs induced by neuroinflammation after exploratory laparotomy in elderly mice was suggested to be involved in the development of PNDs [29, 30], whereas in nonsurgical models, NMDAR downregulation or hypofunction was reported in acute and chronic neuroinflammation [31–34]. In our previous study, we did not detect any alterations in NMDARs after lipopolysaccharide (LPS)-induced neuroinflammation [19]. Hence, how surgery-triggered neuroinflammation affects NMDARs and whether the changes in NMDAR expression or function mediated by neuroinflammation contribute to the development of PNDs remain poorly understood.

In the present study, we explored the potential mechanisms responsible for the development of PNDs after abdominal surgery in the elderly rats. We aimed to determine whether surgery-induced transient neuroinflammation caused long-lasting changes in NMDARs expression or function, as evidenced by impairments in the learning-dependent structural plasticity of dendritic spines that ultimately lead to persistent PNDs.

## Materials and methods

### Reagents

Antibodies used in this study included the polyclonal rabbit antibodies against NR2B (#4207, Cell Signaling Technology, Beverly, MA, USA), NR1 (#5704, Cell Signaling Technology), GluA1 (#13185, Cell Signaling Technology), GluA2 (#13607, Cell Signaling Technology), GluA3 (#3437, Cell Signaling Technology), GluA4 (#8070, Cell Signaling Technology), PSD-95 (#3450, Cell Signaling Technology), phospho-Cofilin (#3311, Cell Signaling Technology), Cofilin (#5175, Cell Signaling Technology), Arp3 (#4738, Cell Signaling Technology), NR2A (#05-901R, Millipore, Merck KGaA, Darmstadt, Germany), NR2C (#OPA1-04020, Millipore), NR2D (#PA5-87624, Millipore), AKAP150 (#07-210, Millipore), HMGB1 (#10829-1-AP, Proteintech, Rosemont, IL, USA), Calnexin (#10427-2-AP, Proteintech), Synaptophysin (#17785-1-AP, Proteintech), Iba-1 (#019-19741, Wako Chemicals, Osaka, Japan), and the monoclonal mouse antibodies against NR2A (#MA5-27692, Invitrogen, Carlsbad, CA, USA), NR2B (#MA1-2014, Invitrogen), β-actin (#66009-1-Ig, Proteintech), GAPDH (#60004-1-Ig, Proteintech), Syntaxin (#66437-1-Ig, Proteintech), NeuN (#MAB377, Millipore), RhoA (#ARH04, Cytoskeleton Inc., Denver, CO, USA), Rac1 (#ARC03, Cytoskeleton), Cdc42 (#ACD03, Cytoskeleton), and Rac1-GTP (#26903, Neweast Biosciences, Wuhan, China). Pharmacological agents included glycyrrhizin (#50531, Sigma, St. Louis, MO, USA).

### Animals

Eighteen-month**-**old male Sprague–Dawley rats (600–900 g) were obtained from the Animal Center of Chongqing Medical University. Animals were housed in groups of 3 individuals per cage in a standard controlled environment at a constant temperature of 22 ± 2 °C and humidity of 50 ± 10% with food and water available ad libitum. The animal room was maintained on a 12 h light/dark cycle. All animal experiments were approved by the Ethics Committee of Chongqing University Cancer Hospital and were performed in strict accordance with the National Guidelines for the Care and Use of Laboratory Animals.

### Abdominal surgery

Abdominal surgery was performed as previously described [35]. Briefly, rats were anesthetized with sevoflurane (2–3% sevoflurane in O_2_ at 1 L/min) and a single intraperitoneal (i.p.) injection of fentanyl (5 µg/kg) and placed on a heating pad. Following median laparotomy, the intestine was exteriorized and the superior mesenteric artery was dissected and occluded with a microvascular clip for 20 min. After releasing the clip, the intestine was placed back in the peritoneal cavity, the abdominal wall along the incision was infiltrated with 0.25% ropivacaine and the surgical incision was closed with sterile sutures. Rats that did not receive surgery and anesthesia served as controls. Rats that only received sevoflurane and fentanyl served as anesthesia.

### Behavioral tests

Behavioral tests, including object location memory (OLM), object recognition memory (ORM), object-in-place (OIP), Morris water maze (MWM), and open field (OF), were performed in a sound-attenuated room with a controlled light intensity (3 lx) using the methods described previously [19, 36, 37]. Prior to OLM, ORM, and OIP tests, rats were handled for 5 min/day followed by habituation to the experimental apparatus (50 × 50 × 40 cm) for 3 consecutive days in the absence of objects.

**Fig. 1:**
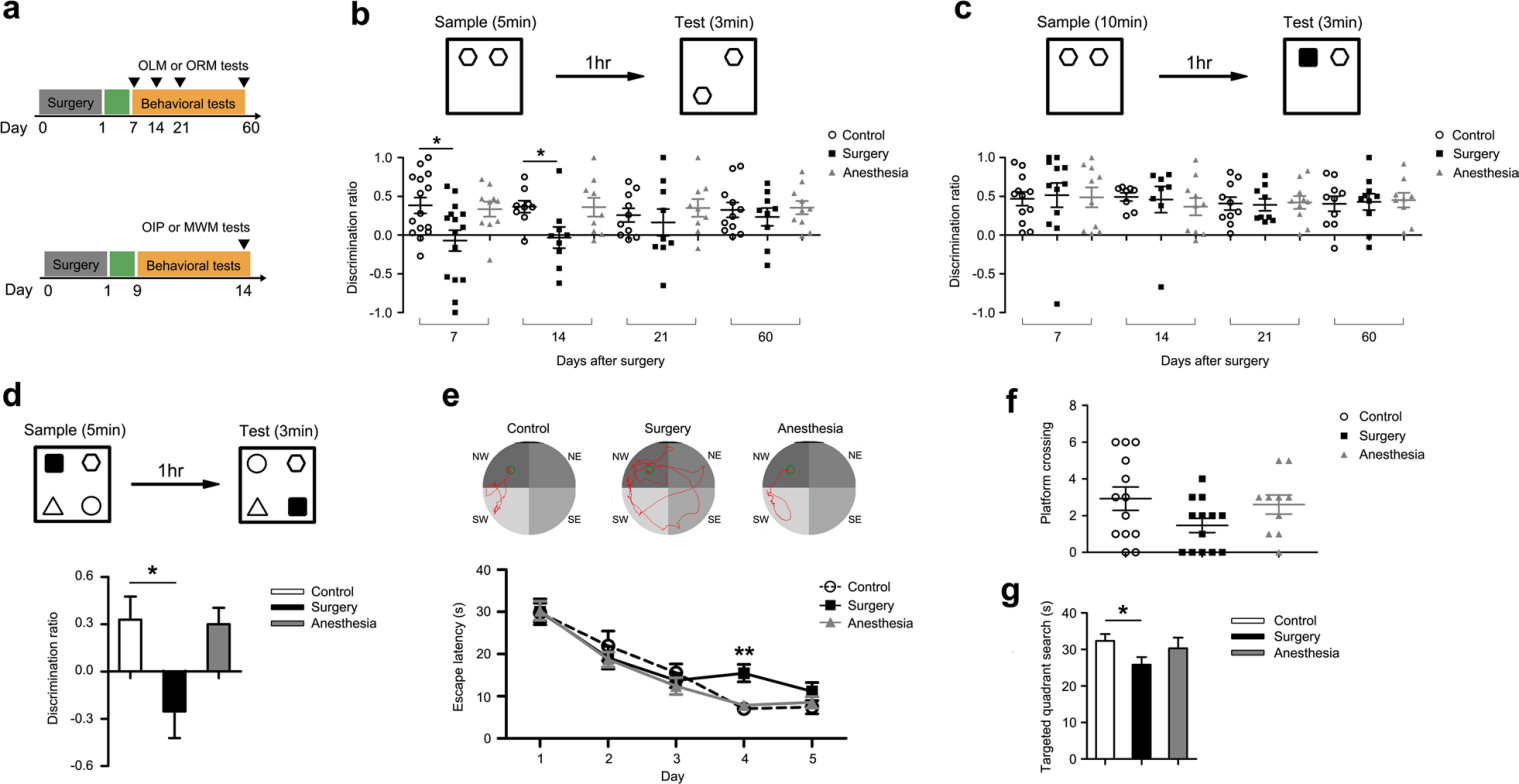
Surgery caused long-term spatial memory deficits. **a** PNDs model was established by performing abdominal surgery and OLM and ORM tests were performed on postoperative days 7, 14, 21, and 60 (top panel). The OIP test was performed on postoperative day 14 and MWM test was performed from postoperative days 9–14 (bottom panel). **b** Experimental procedure for OLM and discrimination ratios at different timepoints (day 7: control, *n* = 15, surgery, *n* = 15, anesthesia, *n* = 10; day 14: control, *n* = 9, surgery, *n* = 9, anesthesia, *n* = 9; day 21: control, *n* = 10, surgery, *n* = 9, anesthesia, *n* = 9; day 60: control, *n* = 11, surgery, *n* = 9, anesthesia, *n* = 10). **c** Experimental procedure for ORM and discrimination ratios (day 7: control, *n* = 12, surgery, *n* = 12, anesthesia, *n* = 10; day 14: control, *n* = 8, surgery, *n* = 8, anesthesia, *n* = 10; day 21: control, *n* = 10, surgery, *n* = 9, anesthesia, *n* = 10; day 60: control, *n* = 10, surgery, *n* = 10, anesthesia, *n* = 9). **d** Experimental procedure for OIP and discrimination ratio on postoperative day 14 (control, *n* = 8, surgery, *n* = 10, anesthesia, *n* = 10). **e** Representative swimming paths during the acquisition session of the MWM and escape latency from postoperative days 9–13. The number of platform crossings **f** and time spent in the target quadrant **g** during the probe trial session of the MWM on postoperative day 14 (control, *n* = 13, surgery, *n* = 13, Anesthesia, *n* = 10). *OLM* object location memory, *ORM* object recognition memory, *OIP* object-in-place, *MWM* Morris water maze. Data are presented as means ± SEM and statistical analyzed by a two-way ANOVA followed by a Tukey’s test (**b** and **c**), a one-way ANOVA followed by a Tukey’s test (**d** and **g**), a Kruskal–Walls *H* test (**f**), or a repeated ANOVA followed by a Tukey’s test (**e**). *p < 0.05 and **p < 0.01 compared with Control

The OLM test comprised two sessions. In the sample session, rats were placed in the apparatus containing two identical objects and allowed to freely explore them for 5 min. After a 1-h delay, one object was moved to a novel location, while the other object remained in the original location (Fig. 1b). Rats were returned to the apparatus and allowed 3 min to explore (test session). Similarly, in the ORM sample session, rats were allowed to freely explore the two identical objects for 5 min. One hour later, one of the objects was replaced with a novel object, and rats were given 3 min of exploration (test session, Fig. 1c). In the OIP test, four different objects were placed in the four corners of the apparatus, and rats were allowed 5 min to explore them (sample session). After 1 h, rats were again placed in the apparatus, but the locations of two of the objects were exchanged, while the other two objects remained at the original locations (Fig. 1d). Rats were allowed to explore the objects for 3 min in the test session. All behaviors in the sample and test sessions were video recorded and analyzed by a researcher blinded to the experimental design. Object exploration was defined as rats directing the nose at a distance of less than 1 cm away from the object. The exploration times were calculated as a discrimination ratio (DR, DR = (T_novel_-T_familiar_)/(T_novel_ + T_familiar_) or DR = (T_displaced_-T_stationary_)/(T_displaced_ + T_stationary_)). Rats that explored the objects for less than 3 s total during either the sample or test session and exhibited an object preference during the sample session (DR > 0.2) were excluded from further analysis according to the criteria established in a previous study [37].

In the MWM test, the hidden platform was placed in a black circular tank (150 cm in diameter) filled with water (22 ± 2 °C) containing ink. The swimming path of each rat was recorded by a video camera mounted directly above the tank. The MWM test consisted of two sessions: the acquisition session for five consecutive days and the probe trial session on day 6. In the acquisition session, rats performed four trials per day with a 30 s interval, and the escape latency in each trial was recorded. In each trial, rats were placed in the tank at a random starting position and allowed to search for the platform within 60 s. If rats failed to find the platform within 60 s, they were guided to the platform and allowed to stay on it for 10 s, then the escape latency was recorded as 60 s. For the probe trial session, a single trial in which the original platform was removed was conducted 24 h after the last trial of the acquisition session. Rats were placed in the opposite quadrant to the platform quadrant and allowed to swim for 60 s. The number of platform crossings and time spent in the targeted quadrant were recorded.

In the OF test, rats were placed in the center of a chamber (100 × 100 × 50 cm) and allowed to explore the arena for 5 min. Spontaneous activity was recorded by a video tracking system, and the total distance traveled and time spent in the center area were measured automatically by OF motion–detection software (Zhenghua Biotech Co., Ltd., Huaibei, China).

### Enzyme-linked immunosorbent assay (ELISA)

Commercially available ELISA kits for measuring serum HMGB1 (#SEKR-0074, Solarbio Science & Technology Co., Ltd., Beijing, China), interleukin-1β (IL-1β, #PRTA00, R&D System, Minneapolis, MN, USA), IL-6 (#PR6000B, R&D System), and tumor necrosis factor-α (TNF-α, #PRLB00, R&D System) levels and dorsal hippocampal IL-1β, IL-6, and TNF-α levels were used according to the manufacturers’ instructions. Briefly, blood samples were collected transcardially under anesthesia with an overdose of sevoflurane (5% for 5 min) and centrifuged at 3,000 g for 10 min at 4 °C; then, the serum was collected. Concurrently, the dorsal hippocampus (dHPC) was dissected based on the atlas of Paxinos and Watson [38] and homogenized in radioimmunoprecipitation (RIPA) lysis buffer followed by centrifugation at 12,000*g* for 10 min at 4 °C. The supernatant was collected, and the protein concentration was determined using a bicinchoninic acid (BCA) assay kit (Beyotime Biotechnology, Shanghai, China). All serum and tissue samples were used to detect the concentrations of HMGB1 and proinflammatory cytokines, and the absorbance was quickly read using an ELISA microplate reader as described in our previous study [39].

### Subcellular fractionation

The synaptosomal membrane (SM) and synaptic plasma membrane (SPM) fractions were prepared using a previously described procedure [40]. The dHPC tissues were homogenized by sonication in homogenization buffer (0.32 M sucrose, 0.5 mM MgSO_4_, 0.1 mM EGTA, and 10 mM HEPES, pH 7.4). Then, the homogenate (H) was centrifuged at 1,000 g for 10 min to remove nuclei and cell debris, and the supernatant was centrifuged at 13,000 g for 10 min to obtain the crude synaptosome pellet. Next, the crude synaptosome was separated by Ficoll density gradient (8% and 14% w/v) centrifugation at 63,000*g* for 50 min. The synaptosome fraction was located at the interface of the 8 and 14% Ficoll solutions. Then, the synaptosome pellets were resuspended and centrifuged at 145,000*g* for 20 min to obtain the isolated SM as the pellet. The SM was lysed in hypotonic lysis buffer (3 mM Tris–HCl, pH 8.5) for 20 min with rotation and then centrifuged at 45,000*g* for 15 min to produce the crude SPM. The crude SPM was resuspended in a 34% sucrose solution and subjected to another Ficoll density gradient (10% and 28.5%) centrifugation step at 90,000*g* for 35 min. The purified SPM fraction was obtained at the interface between the 28.5% and 34% Ficoll solutions.

Separation of the synaptic and extrasynaptic membrane fractions was performed as described in previous studies by ours and other groups [19, 41]. Briefly, the crude synaptosomal pellet was separated by 0.85/1.0/1.2 M sucrose density gradient centrifugation (825,000*g* for 2 h). The synaptosomes were obtained from the 1.0/1.2 M sucrose interface, and synaptosomal pellets were subsequently resuspended in buffer containing 0.5% Triton X-100 and 20 mM HEPES (pH 7.2). Then, the suspension was incubated for 30 min with gentle rotation followed by centrifugation at 320,000 g for 20 min. The pellet, TxP (Triton X-100 insoluble), was defined as the postsynaptic density (PSD)-associated (synaptic) fraction, and the supernatant, TxS (Triton X-100 soluble), was defined as the extrasynaptic fraction. All procedures were performed at 4 °C, and all buffers contained protease inhibitor cocktails. Protein concentrations in all fractions were determined using the BCA assay and adjusted for western blotting.

### Western blotting

The microdissected dHPC, ventral hippocampus (vHPC), prefrontal cortex (PFC), and perirhinal cortex (PRH) tissues were lysed in RIPA lysis buffer containing a mixture of protease and protein phosphatase inhibitors and centrifuged at 12,000*g* for 10 min at 4 °C. The supernatants were then assayed for total protein concentrations using the BCA assay kit. Each sample containing 10 μg of protein was separated on an 8% or 15% SDS–PAGE gel. Next, proteins were transferred to a 0.22 μm polyvinylidene fluoride membrane and blocked with Tris-buffered saline with Tween 20 (TBST) containing 5% milk or 3% bovine serum albumin (BSA) for 1 h at room temperature. The blots were incubated with primary antibodies against NR2A (1:1000), NR2B (1:1000), NR1 (1:1000), NR2C (1:1000), NR2D (1:1000), GluA1 (1:1000), GluA2 (1:1000), GluA3 (1:1000), GluA4 (1:1000), PSD-95 (1:1000), Syntaxin (1:1000), AKAP150 (1:1000), HMGB1 (1:1000), Arp3 (1:1000), Rac1 (1:1000), Cdc42 (1:500), RhoA (1:500), phospho-Cofilin (1:1000), Cofilin (1:1000), Calnexin (1:5000), Synaptophysin (1:10,000), GAPDH (1:50,000) or β-actin (1:10,000) overnight at 4 °C and then incubated with goat anti-rabbit IgG–HRP (1:5000) or goat anti-mouse IgG–HRP (1:5000) for 1 h at room temperature. The protein bands were detected using enhanced chemiluminescence reagents and photographed. For stripping and reprobing, membranes were washed with TBST and incubated with stripping buffer (#P0025N, Beyotime, Shanghai, China) for 1 h at room temperature.

### Pull-down assays

The activities of Rac1, Cdc42, and RhoA GTPases in the dHPC were measured using a Rho/Rac1/Cdc42 (#BK030, Cytoskeleton) pull-down activation assay kit as previously described [42]. As the RBD region of Rhotekin has a high affinity for GTP–Rho (active form of RhoA), and the PBD region of PAK has a high affinity for both GTP–Rac1 (active form of Rac1) and GTP–Cdc42 (active form of Cdc42). The Rhotekin–RBD and PAK–PBD affinity beads supplied in this kit can bind specifically to the active form of the GTPases that can be pulled-down in the bead pellet. Briefly, the isolated dHPC was ground in liquid nitrogen and homogenized in cell lysis buffer containing protease inhibitors. Large debris was removed by centrifugation at 12,000*g* for 10 min at 4 °C. The supernatants containing 800 μg of total protein were incubated with 15 μg of PAK–PBD beads or 50 μg of Rhotekin–RBD beads on a rotator for 1 h at 4 °C. The protein extracts were separated on 15% SDS–PAGE gel for western blotting with the following primary antibodies supplied in the kit: Rac1 (1:500), Cdc42 (1:250), and RhoA (1:500).

### Histology and immunohistochemistry

Rats were deeply anesthetized with sevoflurane and transcardially perfused with phosphate-buffered saline (PBS), followed by 4% paraformaldehyde (PFA). Brains were extracted and postfixed with 4% PFA for 12 h at room temperature and then sectioned into 50 μm coronal slices using a vibratome (VT1000, Leica, Germany). For the histological analysis, sections containing the dHPC (− 2.5–− 4 AP) were mounted on glass slides and dried. Sections were stained with 1% cresyl violet for 30 min at 37 °C, dehydrated with a series of graded ethanol solutions, cleared with xylene, and coverslipped as previously described [43]. Nissl staining within the dHPC was quantified in images captured at 400 × magnification with a Zeiss AX10 microscope coupled to a Zeiss AxioCam ICc 5 digital camera. For immunohistochemistry, sections were blocked with PBS containing 5% goat serum and 0.4% Triton X-100 for 2 h at room temperature and then incubated with primary antibodies against HMGB1 (1:500), Iba-1 (1:1000), NR2A (1:500), NR2B (1:500), Synaptophysin (1:3000), PSD-95 (1:500), Rac1-GTP (1:500) or NeuN (1:5000) for 48 h at 4 °C. Finally, sections underwent three washes for 10 min each, followed by a 2 h incubation with the following secondary antibodies: Alexa Fluor 488-conjugated goat anti-rabbit IgG (1:500), Alexa Fluor 549-conjugated goat anti-rabbit IgG (1:500), Alexa Fluor 488-conjugated goat anti-mouse IgG (1:500), Alexa Fluor 405-conjugated goat anti-mouse IgG (1:500), or HRP-conjugated goat anti-mouse IgG (1:1000). The sections used to detect the NeuN levels were stained with 3,3’-diaminobenzidine (DAB) solution after an incubation with the secondary antibody. Images were acquired with a Zeiss LSM 800 confocal microscope or Zeiss AX10 microscope. All images were analyzed using FIJI software (National Institutes of Health, Bethesda, MD, USA).

### Microglial morphological analysis

Multiple Z-stack confocal images of microglia in the dHPC were acquired at 1 μm intervals using a 63 × objective. Consecutive Z-stack images were reconstructed and converted to a maximum intensity two-dimensional (2D) projection, and then a binary mask was generated by thresholding the 2D projection of microglia. The process length and cell body area of each microglia were measured using FIJI software. Sholl analysis was used to evaluate changes in the microglial morphology by measuring the number of intersections between microglial processes and each increasing circle to create a Sholl plot [44, 45]. The concentric circles started from the cell soma with a radius step size of 10 μm. Six Iba-1-positive cells in the same fields of view in the stratum radiatum of dHPC were chosen per section, and four consecutive sections containing the dHPC (-2.5 ~ -4 AP) were analyzed per animal (four animals per group).

### Golgi staining

Golgi staining was conducted using a GolgiCox OptimStain Kit (#HTKNS1125, Hitobiotec, Kingsport, TN, USA) as previously described [46]. For this experiment, the anesthetized rats were rapidly sacrificed. Brains were removed, rinsed with distilled water, and immersed in Golgi impregnation solution in the dark at room temperature for 2 weeks. Afterward, brains were transferred into another solution provided with the kit and incubated in the dark for 48 h at 4 °C. After completing the impregnation, brains were sectioned into 200 μm coronal slices with a vibratome. Finally, the slices were stained according to the manufacturers’ instructions, dehydrated, cleared, and coverslipped.

The dendrite complexity of each pyramidal neuron was analyzed using the Sholl analysis described above. Multiple Z-stack images of dorsal hippocampal CA1 pyramidal neurons were captured with a Zeiss AX10 microscope (20 × objective), and at least 24 neurons from each group were used. Neurons to be traced in the CA1 region were selected using predetermined criteria. Neurons were fully impregnated and relatively isolated from neighboring cells [47]. Neurons were traced using the Simple Neurite Tracer Plugin in FIJI software beginning at the cell soma. Apical and basal dendrites were traced in their entirety. For each neuron, the number of dendrites and total dendritic length were calculated. The number of intersections was analyzed separately for apical and basal dendrites.

For the spine density analysis, images of dendritic spines from the secondary and tertiary branches of apical dendrites of dorsal hippocampal CA1 pyramidal neurons were captured under 1000 × magnification (oil immersion objective). Spine density was calculated by quantifying the number of spines per 30 μm of dendrite length. Dendritic spines are classified into four shape-based categories: 1) mushroom spines with large heads and short necks, 2) thin spines with small heads and long necks, 3) filopodia spines with long, thin dendritic protrusions and without clear heads, and 4) stubby spines without necks [48].

### Statistical analysis

Statistical analyses were performed with SPSS (version 22, IBM, New York, NY, USA). Normality and homogeneity of variance were detected using Kolmogorov–Smirnov and Levene’s tests, respectively, and data are presented as the means ± SEM. One-way ANOVA, two-way ANOVA, repeated ANOVA or Student’s t test was used to evaluate the differences in means values among the groups, as appropriate, followed by Tukey’s post hoc analysis. A significant difference was considered at the level of a *p* value less than 0.05.

## Results

### Spatial memory was impaired after surgery

To investigate the consequences of surgery on long-term cognition, postoperative cognitive function was measured using the OLM and ORM tasks at different timepoints (Fig. 1a, top). Rats in the surgery group had significant deficits in OLM on days 7 and 14 following surgery compared with nonsurgical controls. Rats that underwent surgery were unable to distinguish the displaced object from the stationary object, as reflected in the negative DR. The recognition impairment gradually recovered and almost returned to the control level on postoperative day 60 (Fig. 1b). In contrast, ORM was not impaired in rats that underwent surgery at each timepoint, and their DRs were similar to those of control rats (Fig. 1c). No behavioral changes were detected in rats only received anesthesia either in the OLM or ORM tasks (Fig. 1b, c). The poor performance of rats that underwent surgery in the OLM was not due to a lack of interest, as rats in both groups explored the objects at similar levels during sample and test sessions (Additional file 1: Table S1). In addition, we excluded the possibility that this impairment was associated with an effect of anxiety by assessing rats’ behavior in the OF (Additional file 1: Fig. S1). To exclude the effect of superior mesenteric artery clamping as a confounding factor on the observed surgery-induced spatial recognition memory deficit, we established an additional group of aged rats that only received anesthesia and laparotomy by exposing the superior mesenteric artery for 20 min but without clamping. Rats in this sham surgery group also exhibited significantly impaired memory in the OLM compared with control rats on postoperative day 14 (Additional file 1: Fig. S2). Subsequently, we then used a slightly more complex behavioral paradigm, OIP, a task that involves the integration of both object and spatial information to further ensure the selective spatial recognition memory deficit [49]. We used an independent set of rats for the OIP test on postoperative day 14 to exclude the potential perturbations of OLM and ORM on the behavior in the OIP (Fig. 1a, bottom). As expected, rats in the surgery group did not exhibit a displaced object preference as shown by a significantly decreased DR (Fig. 1d). The impairment was also not due to a lack of interest in object exploration (Additional file 1: Fig. S3a). We still did not find any behavioral changes in rats only received anesthesia in the OIP test (Fig. 1d). Since recognition memory involving spatial information mainly depends on the hippocampus, especially the dHPC [49, 50], and the dHPC concurrently plays a vital role in spatial navigation, we asked whether the spatial navigation ability was damaged after surgery. Accordingly, the MWM task which is recognized as one of the most classical spatial navigation paradigms, was performed (Fig. 1a, bottom). We discovered that rats that underwent surgery were much slower at learning to reach the hidden platform than the control rats on the fourth day of the learning trial (Fig. 1e). In the probe trial in which the platform was removed, operated rats crossed the platform location less frequently (Fig. 1f) and spent less time in the target quadrant than the control rats (Fig. 1g). No motor dysfunction was detected by evaluating the swimming speed (Additional file 1: Fig. S3b). No behavioral changes in rats only received anesthesia in the MWM task (Fig. 1e–g). These results together indicate that elderly rats may develop PNDs relatively later after abdominal surgery and that spatial memory is extremely sensitive in response to surgical trauma.

### Surgery induced an acute systemic inflammatory response and neuroinflammation

**Fig. 2:**
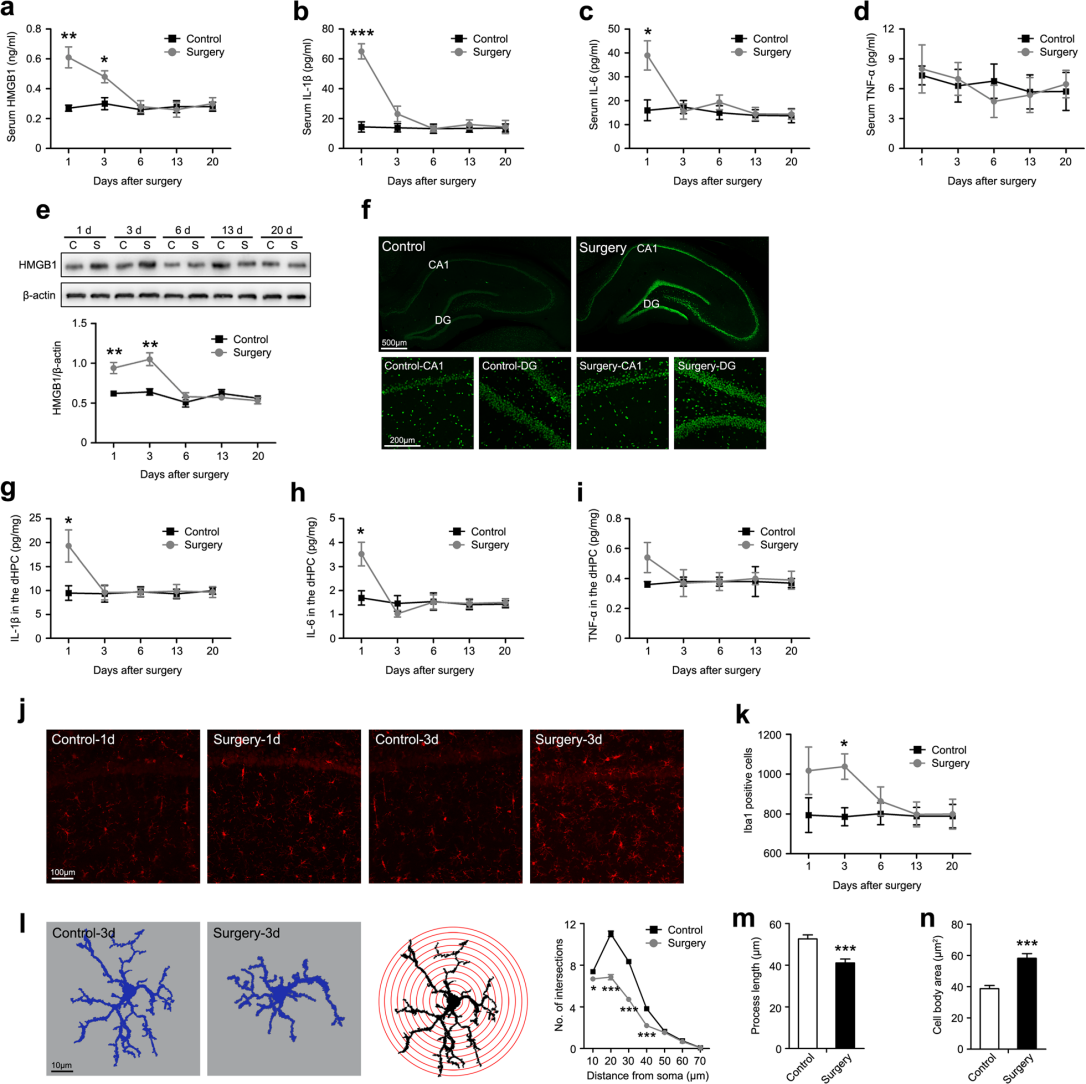
Surgery induced neuroinflammation by triggering the peripheral inflammatory response. The concentration of **a** HMGB1, **b** IL-1β, **c** IL-6, and **d** TNF-α in serum were measured using ELISAs on postoperative days 1, 3, 6, 13, and 20 (*n* = 5 rats per group). **e** Representative immunoblot showing the levels of the HMGB1 protein (top panel) and densitometry analysis of the HMGB1 protein (bottom panel) in the dHPC on postoperative days 1, 3, 6, 13, and 20 (*n* = 5 rats per group). **f** Representative confocal images of immunohistochemical staining for HMGB1^+^ cells (green) in the dHPC captured with a 10 × objective on postoperative day 3. The concentration of **g** IL-1β, **h** IL-6, and **i** TNF-α in the dHPC were measured using ELISAs on postoperative days 1, 3, 6, 13, and 20 (*n* = 5 rats per group). **j** Representative confocal images of immunohistochemical staining for Iba1^+^ cells (red) in the dHPC captured with a 10 × objective on postoperative days 1 and 3. **k** Quantitative analysis of Iba1^+^ cells in the dHPC on postoperative days 1, 3, 6, 13, and 20 (*n* = 5 rats per group). **l** Representative 2D projection images of microglia captured with a 63 × objective (left panel). A schematic diagram of a 2D Sholl grid of concentric spheres beginning from the geometric center of the microglial cell soma (middle panel). Quantitative Sholl analysis of microglial processes by counting the number of intersections (right panel). **m** Total process lengths and **n** cell body area observed in confocal images of immunohistochemical staining for microglia were analyzed using FIJI software (*n* = 96 microglial cells from 4 rats per group). *C* control, *S* surgery. Data are presented as means ± SEM and statistical analyzed by a two-way ANOVA followed by a Tukey’s test (**a**, **b**, **c**, **d**, **e**, **g**, **h**, **i**, and **k**), or a Student’s *t* test (**l**, **m**, and **n**). *p < 0.05 and **p < 0.01 compared with Control

To further investigate the dynamic process of inflammatory changes after surgery, we first measured the serum HMGB1, IL-1β, IL-6, and TNF-α levels from 1 to 20 days postoperatively. Compared with the control rats, the serum HMGB1 levels were significantly increased in rats subjected to surgery on postoperative days 1 and 3 and returned to the control level on day 6 (Fig. 2a). Similarly, the serum IL-1β and IL-6 levels in rats that underwent surgery were remarkably increased on day 1 and generally returned to control levels on day 3 after surgery (Fig. 2b, c). No changes in serum TNF-α levels were observed within 20 days postoperatively (Fig. 2d). Next, we evaluated neuroinflammation in the dHPC after surgery by detecting the expression of the HMGB1 protein and proinflammatory cytokines. HMGB1 protein levels were increased in the surgery group on day 1 and remained elevated up to day 3 postoperatively (Fig. 2e). This significantly elevated HMGB1 expression on postoperative day 3 was confirmed by immunohistochemistry (Fig. 2f). In addition, the significant upregulation of IL-1β and IL-6 in the dHPC of rats that underwent surgery was also detected on postoperative day 1, whereas a slightly increased levels of TNF-α in the operated rats was observed (Fig. 2g–i). Since microglia are the key innate immune cells in the CNS and are activated quickly in response to environmental changes in the brain [51], we focused specifically on microglia in the dHPC and investigated the microglial response to surgical trauma. First, we counted the number of Iba-1^+^ (a microglia marker) cells and found that surgery induced an increase in the number of microglia from postoperative days 1–3 (Fig. 2j, k). Then, we performed a detailed quantitative morphometric analysis of microglia, because microglial activation is frequently accompanied by morphological changes. As shown in the 2D projection images, microglia in the operated rats exhibited shorter, thicker, and sparsely branched processes (Fig. 2l, left). Affirming this observation, Sholl analysis showed that the operated rats had fewer microglial process intersections than the control rats on postoperative day 3 (Fig. 2l, right). We also assessed activated microglia by quantifying their shapes using a skeleton analysis. Microglia were activated after surgery, with retracted processes and an enlarged cell body (Fig. 2m, n). Taken together, these data indicate that surgery induces transient systemic inflammatory responses and neuroinflammation.

### Surgery-induced neuroinflammation caused persistent reductions in the synaptic levels of the NR2A and NR2B subunits

**Fig. 3:**
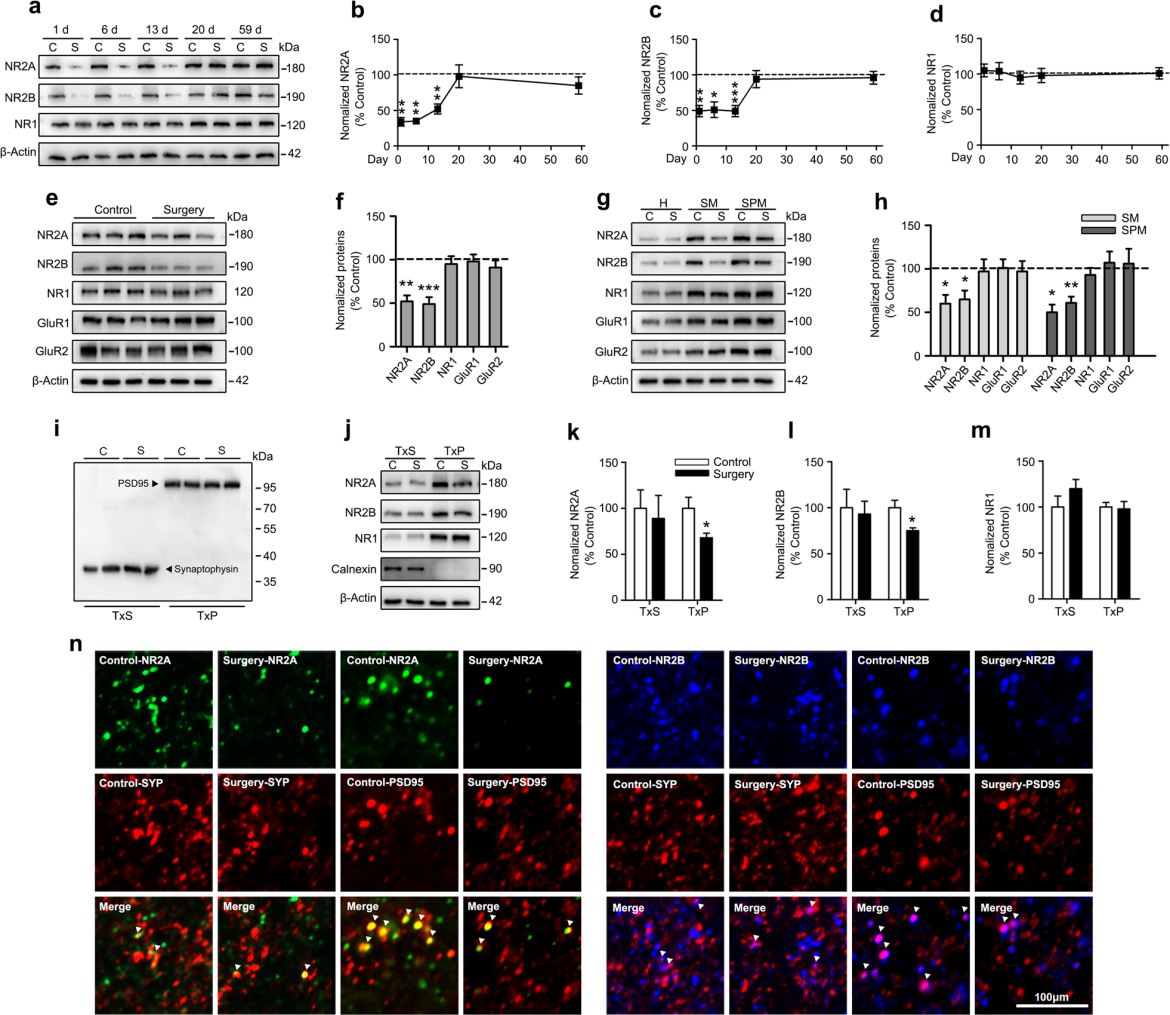
Surgery caused a selective reduction in the levels of synaptic NR2A and NR2B subunits. **a** Representative immunoblots and densitometry analysis of **b** NR2A, **c** NR2B, and **d** NR1 proteins levels in the dHPC on postoperative days 1, 6, 13, 20, and 59 (*n* = 6 rats per group). **e** Representative immunoblots and **f** densitometry analysis of NR2A, NR2B, NR1, GluA1, and GluA2 proteins levels in the dHPC on postoperative day 13 (*n* = 6 rats per group). **g** Representative immunoblots showing levels of the NR2A, NR2B, and NR1 proteins in the total dHPC H, SM, and SPM. **h** Densitometry analysis of NR2A, NR2B, and NR1 proteins levels in the SM and SPM (*n* = 6 rats per group). Representative immunoblots showing levels of **i** the PSD-95, Synaptophysin, and **j** Calnexin indicated the efficiency of separating TxP (synaptic) and TxS (extrasynaptic) fractions of the dHPC. **j** Representative immunoblots and densitometry analysis of (k) NR2A, **l** NR2B, and **m** NR1 proteins levels in the TxS and TxP fractions of the dHPC on postoperative day 13 (*n* = 6 rats per group). **n** Representative confocal images of double staining for NR2A (green puncta) at the extrasynaptic (colocalized with synaptophysin^+^ red puncta) and synaptic (colocalized with PSD-95^+^ red puncta) sites (left panel) and NR2B (blue puncta) at the extrasynaptic (colocalized with synaptophysin^+^ red puncta) and synaptic (colocalized with PSD-95^+^ red puncta) sites (right panel) captured with a 63 × objective. *C* control, *S* surgery, *H* homogenate, *SM* synaptosomal membrane, *SPM* synaptic plasma membrane, *SYP* Synaptophysin, *TxS* Triton X-100-soluble, *TxP* Triton X-100-insoluble. Data are presented as means ± SEM and statistical analyzed by a two-way ANOVA followed by a Tukey’s test (**b**, **c**, and **d**), a Student’s *t* test (**f**, **h**, **k**, **l**, and **m**). *p < 0.05, **p < 0.01, and ***p < 0.001 compared with Control

Glutamate NMDARs are highly concentrated in the dHPC, activation of these receptors is known to play an important role in spatial memory [20, 52], and neuroinflammation has been proven to be strongly related to NMDAR downregulation and hypofunctionv [31–33]. Our observation that aged rats presented a selective spatial memory deficit after abdominal surgery along with significant neuroinflammation within the dHPC prompted us to speculate that the changes in NMDAR expression in the dHPC mediated by neuroinflammation may contribute to the development of PNDs. We first detected the levels of NMDAR subunits in the dHPC at different timepoints postoperatively and discovered that significantly reduced levels of NR2A and NR2B in operated rats from postoperative days 1–13 (Fig. 3a–c). No changes in NR1 expression between the two groups were detected at each timepoint (Fig. 3a, d). These results suggest that transient neuroinflammation induced by surgery may downregulate the NR2A and NR2B levels in the dHPC.

Next, we focused on the changes of NR2A and NR2B levels on postoperative day 13, because our results showed that the persistent downregulation of NR2A and NR2B in the dHPC after transient neuroinflammation were temporally associated with long-lasting spatial memory deficits (Fig. 3e, f). Meanwhile, the total levels of α-amino-3-hydroxy-5-methyl-4-isoxazole propionic acid receptor (AMPAR) subunits including GluA1 and GluA2 were not altered in response to surgery-induced neuroinflammation, indicating that NR2A and NR2B were sensitive to neuroinflammation (Fig. 3e, f). We still screened other NMDAR and AMPAR subunits for their responses to neuroinflammation triggered by surgery. In normal adult rodents, NR1, NR2A, NR2B, GluA1, and GluA2 are expressed at high levels in the hippocampus. NR2C, NR2D, GluA3, and GluA4 are expressed at relatively low levels, whereas NR3A and NR3B proteins are almost undetectable [24, 53]. Here, the total levels of NR2C, NR2D, GluA3, and GluA4 in the dHPC were not changed between the groups (Additional file 1: Fig. S4a, b). Surgery-induced neuroinflammation did not alter the expression of presynaptic proteins, including Syntaxin (a presynaptic plasma membrane protein) and Synaptophysin (a presynaptic vesicle membrane protein). In addition, neuroinflammation did not affect the expression of postsynaptic proteins, including a-kinase anchoring protein 150 (AKAP150, an AMPA receptor-associated scaffold protein) and PSD-95 (an NMDA receptor-associated scaffold protein) (Additional file 1: Fig. S4c, d). Moreover, NR2A and NR2B proteins are concentrated in other brain regions, including the vHPC, PFC, and PRH, which are involved in learning and memory [36, 54]. However, no changes in the expression of NR2A, NR2B, and NR1 were observed in these regions (Additional file 1: Fig. S4e–h), suggesting that downregulation of NR2A and NR2B after surgery-induced neuroinflammation appeared to be specific to the dHPC. We next examined whether the specific reductions in the expression of NR2A and NR2B subunits in the dHPC resulted from neuronal loss. Interestingly, the number of neurons in the dHPC of operated rats remained unchanged on postoperative day 13 (Additional file 1: Fig. S4i). Furthermore, no change in the number of mature neurons (NeuN^+^ cells) was observed in different dorsal hippocampal subregions between the two groups (Additional file 1: Fig. S4j).

After discovering the reductions in NR2A and NR2B levels in total protein homogenates of the dHPC, we then asked whether the subcellular localization of NR2A or NR2B was affected after surgery. Notably, surgery-induced neuroinflammation significantly reduced NR2A and NR2B levels in the SM and SPM fractions on postoperative day 13, whereas NR1 levels were not changed. Meanwhile, neuroinflammation did not alter GluA1 and GluA2 levels in either SM or SPM fractions (Fig. 3g, h). Next, we attempted to isolate extrasynaptic (TxS) and synaptic (TxP) fractions by digesting the SM with Triton X-100. The purity of the extrasynaptic and synaptic fractions was initially verified by confirming the unique distribution of Synaptophysin, PSD-95, and Calnexin in distinct subcellular compartments (Fig. 3i, j). As shown in Fig. 3j, all three NMDAR subunits were abundant in the synaptic fractions and were present in the extrasynaptic fractions at low levels. Neuroinflammation reduced synaptic but not extrasynaptic NR2A or NR2B levels on postoperative day 13; however, neither synaptic nor extrasynaptic NR1 proteins levels were altered by neuroinflammation (Fig. 3k–m). To validate this subcellular compartment-specific effect, we conducted immunohistochemistry to investigate the colocalization of NR2A and NR2B subunits with synaptic (PSD-95) or extrasynaptic (Synaptophysin) markers. NR2A expression at the synaptic site was decreased in operated rats, as shown by the lower number of puncta coexpression NR2A and PSD-95 compared to that of the control rats, while extrasynaptic NR2A expression was not altered. Similar results were observed for the expression of synaptic and extrasynaptic NR2B (Fig. 3n).

### Surgery-induced neuroinflammation impaired spine structural plasticity, which was closely associated with NMDAR hypofunction

**Fig. 4:**
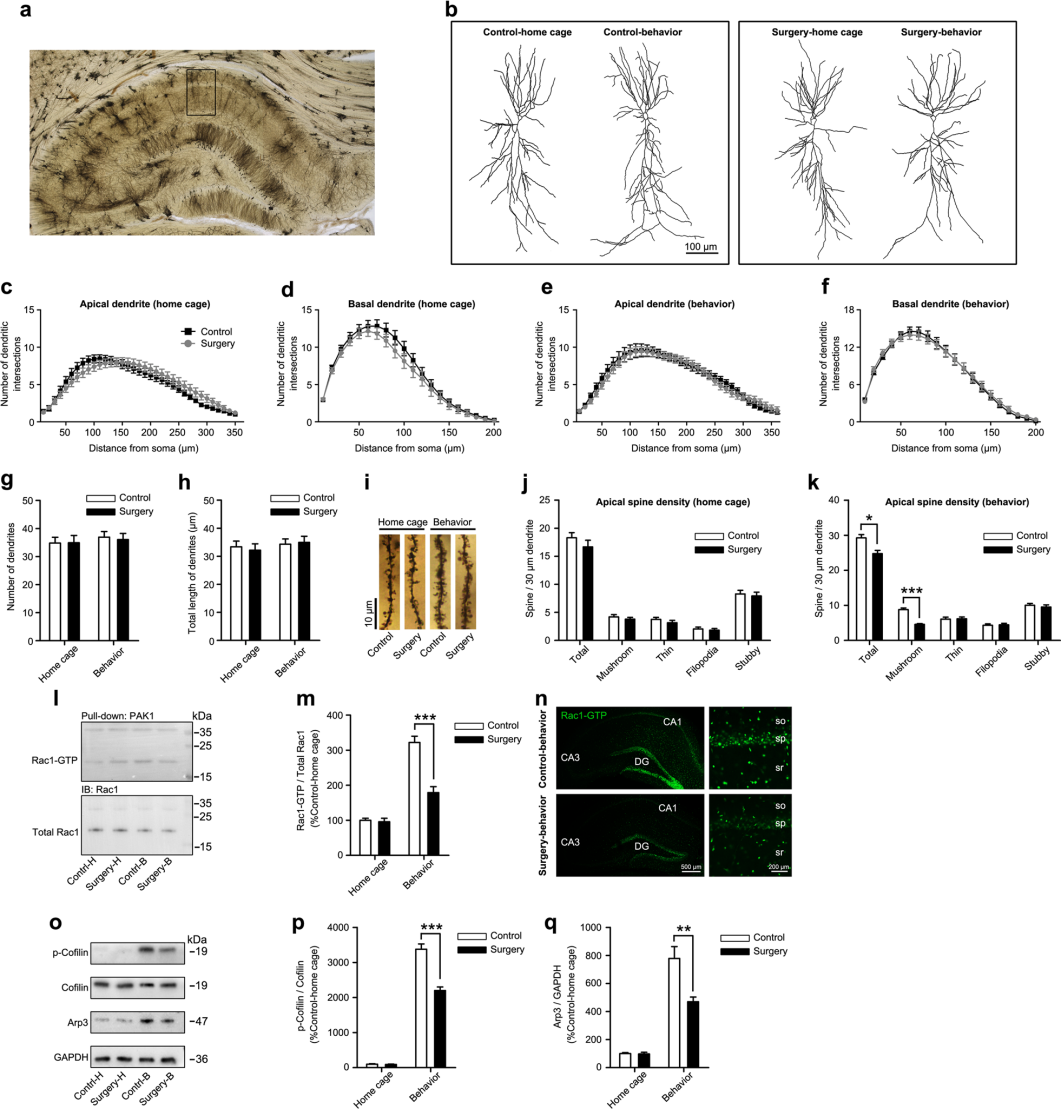
Surgery impaired spine structural plasticity and decreased the activity of Rac1 signaling pathways during learning. **a** Dorsal hippocampal profile image of Golgi staining. **b** Representative tracing images of dorsal hippocampal CA1 pyramidal neurons on postoperative day 14. Sholl analysis of apical and basal dendrites of CA1 pyramidal neurons from rats **c**, **d** without behavioral training (home cage) and **e**, **f** with behavioral training (behavior, n = 24 neurons from 4 rats per group). **g** Total number of dendrites and **h** total dendritic lengths of traced images of dorsal hippocampal CA1 pyramidal neurons were analyzed using FIJI software (*n* = 24 neurons from 4 rats per group). **i** Representative images of the secondary branch of apical dendrites of CA1 pyramidal neurons in the dHPC on postoperative day 14 captured with a 100 × objective. Quantitation of the total spine, mushroom-shaped spine, thin-shaped spine, filopodia-shaped spine, and stubby-shaped spine densities in rats **j** without behavioral training and **k** with behavioral training (n = 24 neurons from 4 rats per group). **l** Representative immunoblots and **m** densitometry analysis of Rac1-GTP (active) and total Rac1 proteins levels in the dHPC on postoperative day 14 (*n* = 6 rats per group). **n** Representative confocal images of immunohistochemical staining for Rac1-GTP^+^ cells (green) in the dHPC captured with a 10 × objective. **o** Representative immunoblots and densitometry analysis of **p** phospho-Cofilin, Cofilin, and **q** Arp3 proteins in the dHPC on postoperative day 14 (*n* = 6 rats per group). *Control-H* control-home cage, *Surgery-H* surgery-home cage, *Control-B* control-behavior, *Surgery-B* surgery-behavior. Data are presented as means ± SEM and statistical analyzed by a Student’s t test (**c**, **d**, **e**, **f**, **j**, and **k**), a one-way ANOVA followed by a Tukey’s test (**g**, **h**, **m**, **p**, and **q**). *p < 0.05, **p < 0.01, and ***p < 0.001 compared with Control

The notable sustained reductions in synaptic NR2A and NR2B protein expression after surgery prompted us to explore whether the function of synaptic NMDARs was also impacted by neuroinflammation. The main function of synaptic NMDARs is to modulate experience-dependent synaptic plasticity during learning and memory, and one important mechanism is the regulation of dendritic structural plasticity, which involves changes in dendritic complexity, dendritic spine density, and size. In normal rodents, reliable changes in hippocampal dendritic structure are observed following spatial-dependent learning experiences [55]. Here, we used another four independent sets of rats, including control-home cage, control-behavior, surgery-home cage, and surgery-behavior, to observe changes in the dendritic structural of CA1 pyramidal neurons in the dHPC after behavioral training. Rats in the control-home cage and surgery-home cage groups received the equivalent treatments as those in the control-behavior and surgery-behavior groups, respectively, but did not undergo the behavioral test. All rats were sacrificed 40 min after behavioral training, and their brains were collected for Golgi staining (Fig. 4a). We first assessed the changes in dendrite complexity using Sholl analysis and did not discover any changes in either apical or basal dendritic complexity among the four groups (Fig. 4b–f). In addition, no significant differences in the total dendritic branching and length were observed between groups (Fig. 4g, h). We then proceeded to examine the density and morphology of dendritic spines located on the secondary or tertiary branches of apical dendrites of the CA1 pyramidal neurons. Without behavioral training, no significant alterations in the total CA1 apical spine density or spine type were observed in the operated rats (surgery-home cage) compared with control rats (control-home cage), indicating that surgery-induced neuroinflammation did not affect the baseline spine density or proportion. The number of total spines and mushroom-shaped spines in behavior**-**trained control rats (control-behavior) was significantly higher than that in control-home cage rats, suggesting that behavioral training itself might stimulate dendritic spine growth and remodeling. Although a similar trend of increase in total spine density was also observed in rats from the surgery-behavior group, the degree of increase was significantly less than that of control-behavior rats. Moreover, the number of mushroom-type spines did not seem to increase in the surgery-behavior group as in the control-behavior group (Fig. 4i–k). Based on these data, experience-dependent dendritic spine structural plasticity regulated by synaptic NMDAR activation is impaired after surgery.

Because the small GTPase Rac1 is a well-established signaling protein that regulates dendritic spine morphogenesis and maturation by modulating the organization of the actin cytoskeleton [56, 57] and Rac1 activity during dendritic spine structural plasticity is mediated by synaptic NMDAR activation [58], we asked whether Rac1 signaling was dysregulated after surgery and causally related to the downregulation of synaptic NR2A and NR2B subunits. Therefore, we performed a pull-down assay using the p21-binding domain of PAK to examine the amount of GTP-bound (active) Rac1. Basal Rac1 activity was not altered after surgery-induced neuroinflammation. Nevertheless, Rac1 activity was significantly increased in control rats that experienced behavioral training, whereas surgery led to a slight increase in the amount of Rac1–GTP after behavioral training. Thus, profound differences in Rac1 activity were detected between the control-behavior and surgery-behavior groups (Fig. 4l, m), and this effect was confirmed by immunohistochemistry for the Rac1–GTP protein (Fig. 4n). We also measured the activities of other small Rho GTPases, but observed no changes in the activation of Cdc42 and RhoA after behavioral training in rats from either the surgery or control groups (Additional file 1: Fig. S5). Next, the level of the actin-related protein 2 and 3 (Arp2/3) subunit Arp3 and the level of phosphorylated Cofilin, which are the two major downstream effectors of Rac1 [59], were detected with or without behavioral training. Notably, surgery significantly decreased the magnitude of the increases in Arp3 and phospho-Cofilin levels after behavioral training (Fig. 4o–q), supporting our hypothesis that surgery-induced neuroinflammation caused hypofunction of synaptic NMDARs by downregulating the expression of synaptic NR2A and NR2B subunits, which impaired experience-dependent dendritic spine structural plasticity by decreasing Rac1 activity and associated downstream signaling pathways.

### Neuroinflammation-induced persistent downregulation of synaptic NR2A and NR2B subunits after surgery contributed to spatial memory deficits by influencing dendritic spine structural plasticity

**Fig. 5:**
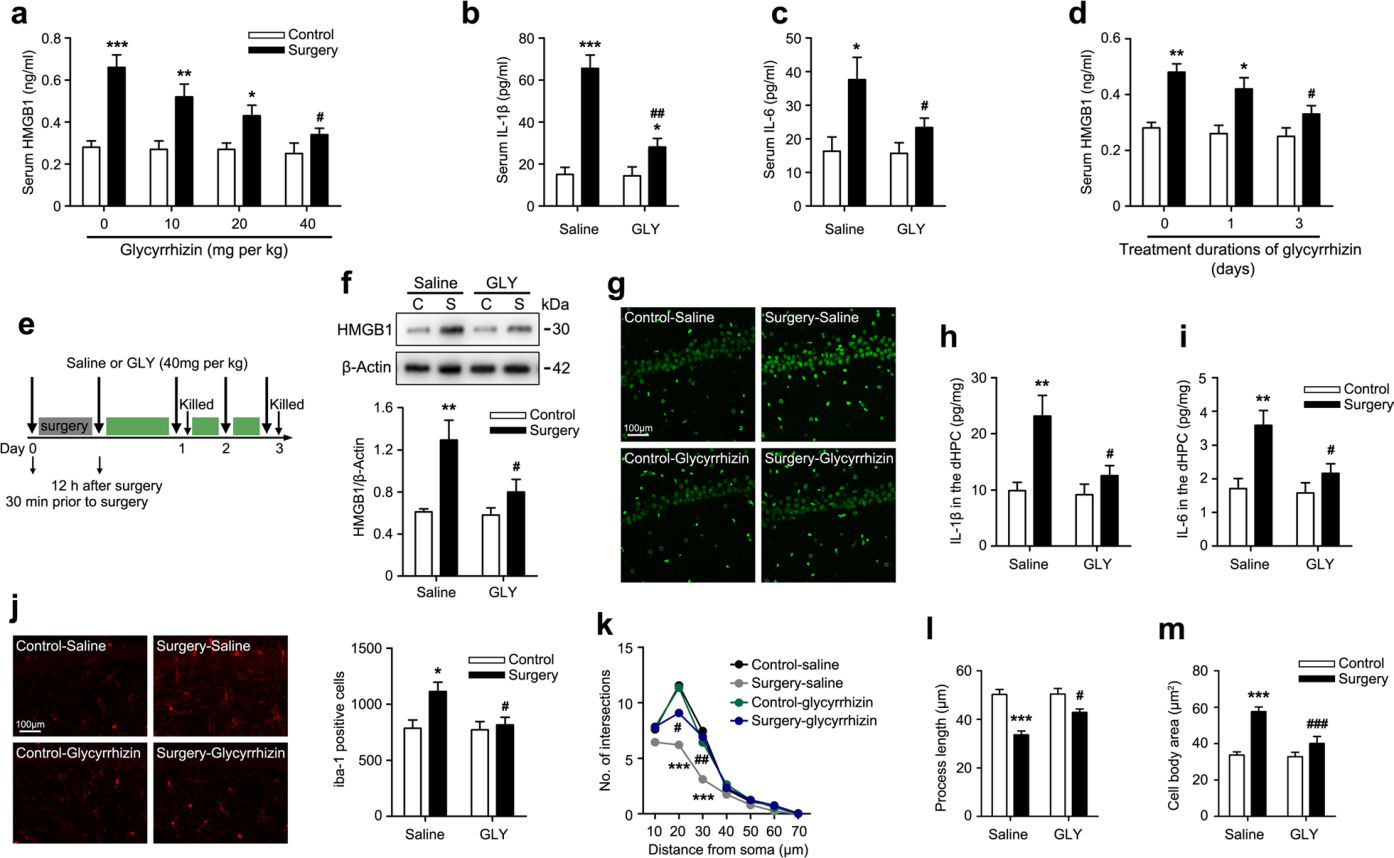
Effects of glycyrrhizin on the systemic inflammatory response and neuroinflammation following surgery. **a** Glycyrrhizin (10, 20, or 40 mg/kg, once) were administered i.p. 30 min before and 12 h after surgery, and the serum HMGB1 concentration was measured using ELISA on postoperative day 1 (*n* = 6 rats per group). The serum **b** IL-1β and **c** IL-6 concentrations were measured using ELISAs on postoperative day 1 (*n* = 6 rats per group). **d** Two modes of glycyrrhizin injections (2 doses: − 30 min, + 12 h; 5 doses: − 30 min, + 12 h, + 24 h, + 48 h, and + 72 h) were administered, and the serum HMGB1 levels were measured using ELISA on postoperative day 3 (*n* = 6 rats per group). **e** Inset of the glycyrrhizin treatment regimen and pharmacological analysis protocol. **f** Representative immunoblots and corresponding densitometry analysis of HMGB1 protein levels in the dHPC on postoperative day 3 (*n* = 6 rats per group). **g** Representative confocal images of immunohistochemical staining for HGMB1^+^ cells in the dHPC on postoperative day 3 captured with a 10 × objective. The concentration of **h** IL-1β and **i** IL-6 in the dHPC were measured using ELISAs on postoperative day 1 (*n* = 6 rats per group). **j** Representative confocal images of immunohistochemical staining for Iba1^+^ cells in the dHPC captured with a 10 × objective (left panel) and quantitative analysis on postoperative day 3 (*n* = 6 rats per group, right panel). **k** Sholl quantitative analysis of microglial process complexity. **l** Total process lengths and **m** cell body area of microglia were analyzed using FIJI software (n = 96 microglial cells from 4 rats per group). GLY: glycyrrhizin, C: control, S: surgery. Data are presented as means ± SEM and statistical analyzed by a Student’s t test (**a**, **d**) or a one-way ANOVA followed by a Tukey’s test (**b**, **c**, **f**, **h**, **i**, **j**, **k**, **l**, and **m**). *p < 0.05, **p < 0.01, and ***p < 0.001 compared with Control-saline; ^#^*p* < 0.05, ^##^*p* < 0.01, and ^###^*p* < 0.001 compared with Surgery-saline

We used glycyrrhizin, a direct inhibitor of HMGB1, to further confirm that the effect of neuroinflammation on spatial memory deficits was mediated by downregulating synaptic NR2A and NR2B subunits. Glycyrrhizin has been reported to inhibit HMGB1 by binding directly to HMGB1 and blocking HMGB1 release [60]. First, different doses of glycyrrhizin (10, 20, or 40 mg/kg once) were administered i.p. 30 min before surgery and 12 h after surgery, and glycyrrhizin dose-dependently inhibited the expression of serum HMGB1 on postoperative day 1, achieving 44% inhibition at 40 mg/kg (Fig. 5a). The effectiveness of this dose was further validated by confirming the reductions in serum IL-1β and IL-6 levels on postoperative day 1 (Fig. 5b, c). Based on the kinetics of HMGB1 accumulation in serum after surgery (Fig. 2a) and the relatively short biological half-life of glycyrrhizin after intravenous administration [61, 62], we reasoned that effective neutralization of the persistent upregulation of serum HMGB1 levels might require repeated dosing. Administration of five glycyrrhizin doses (30 min before surgery and 12 h, 24 h, 48 h, and 72 h after surgery) decreased the serum HMGB1 levels on postoperative day 3 compared with rats without glycyrrhizin treatment or only treated with two doses (- 30 min, + 12 h) (Fig. 5d). Thus, this mode of glycyrrhizin administration in five repeated doses was used in subsequent experiments (Fig. 5e).

We next evaluated the effect of glycyrrhizin treatment on surgery-induced neuroinflammation. Glycyrrhizin reversed HMGB1 overexpression in the dHPC of operated rats on postoperative day 3, whereas saline had no effect (Fig. 5f, g). In addition, increase in IL-1β and IL-6 levels in the dHPC on postoperative day 1 was alleviated by treatment with glycyrrhizin (Fig. 5h, i). Glycyrrhizin alone did not alter the expression of HMGB1, IL-1β, or IL-6 in control rats (Fig. 5f–i). Furthermore, treatment with glycyrrhizin ameliorated the increased microglial density and microglial overactivation in the dHPC on postoperative day 3 (Fig. 5j–m).

**Fig. 6:**
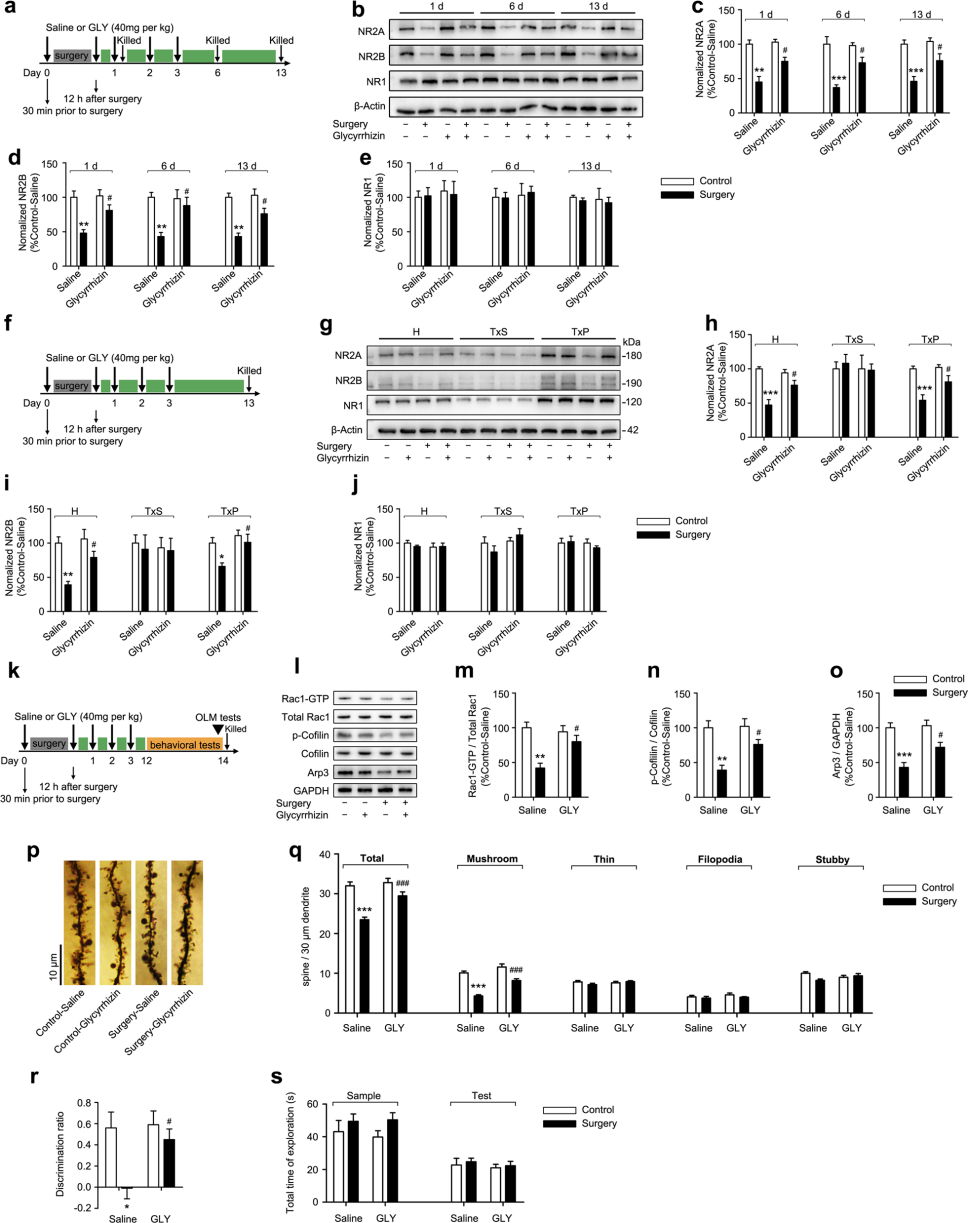
Glycyrrhizin reversed NMDAR downregulation and dysfunction and rescued the spatial recognition memory deficit. **a** Graphic illustration of the experimental protocol. **b** Representative immunoblots and corresponding densitometry analysis of **c** NR2A, **d** NR2B, and **e** NR1 protein levels in the total dHPC homogenate on postoperative days 1, 6, and 13 (n = 6 rats per group). **f** Graphic illustration of the experimental protocol. **g** Representative immunoblots and corresponding densitometry analysis of **h** NR2A, **i** NR2B, and **j** NR1 protein levels in the total dHPC homogenate, TxS, and TxP on postoperative day 13 (*n* = 6 rats per group). **k** Graphic illustration of the experimental protocol. **l**–**o** Representative immunoblots and corresponding densitometry analysis of Rac1–GTP, total Rac1, phospho-Cofilin, Cofilin, and Arp3 protein levels in the dHPC on postoperative day 14 following behavioral training (*n* = 6 rats per group). **p** Representative images of the secondary branch of apical dendrites of CA1 pyramidal neurons in the dHPC on postoperative day 14 captured with a 100 × objective. **q** Quantitative analysis of the total spine, mushroom-shaped spine, thin-shaped spine, filopodia-shaped spine, and stubby-shaped spine densities in the dHPC (*n* = 24 neurons from 4 rats per group). **r** Discrimination ratio and **s** total time spent exploring objects in the OLM during sample and test sessions on postoperative day 14 (Control-saline, *n* = 8, Surgery-saline, *n* = 8, Control-glycyrrhizin, *n* = 9, Surgery-glycyrrhizin, *n* = 10). *GLY* glycyrrhizin, *OLM* object location memory, *H* homogenate, *TxS* Triton X-100-soluble, *TxP* Triton X-100-insoluble. Data are presented as means ± SEM and statistical analyzed by a one-way ANOVA followed by a Tukey’s test (**c**, **d**, **e**, **h**, **i**, **j**, **m**, **n**, **o**, **q**, **r**, and **s**). *p < 0.05, **p < 0.01, and ***p < 0.001 compared with Control-saline; ^#^*p* < 0.05 and ^###^*p* < 0.001 compared with Surgery-saline

Next, we asked whether glycyrrhizin could rescue the persistent downregulation of NR2A and NR2B in the total dHPC protein homogenate postoperatively. Glycyrrhizin treatment remarkably reversed the decreased levels of NR2A and NR2B in operated rats on postoperative days 1, 6, and 13, while the levels of NR1 expression were not altered (Fig. 6a–e). We then further observed that glycyrrhizin treatment significantly rescued the decreased levels of synaptic NR2A and NR2B in operated rats on postoperative day 13, whereas it did not alter NR1 expression in each subcellular fraction (Fig. 6f–j). In addition, glycyrrhizin significantly reversed the decreased Rac1 activity and levels of downstream effectors on postoperative day 14 when operated rats experienced behavioral training (Fig. 6k–o), and the reductions in the total spine and mushroom-shaped spine densities on apical dendrites in the CA1 region of the dHPC after behavioral training were reversed by glycyrrhizin treatment in operated rats (Fig. 6p, q). Finally, glycyrrhizin rescued the spatial memory deficit on postoperative day 14 without affecting the predisposition to explore objects (Fig. 6r, s). These results together confirm that surgery-induced neuroinflammation mediates the downregulation of synaptic NR2A and NR2B subunits, which may be a potential molecular mechanism underlying PNDs development.

## Discussion

As shown in the present study, surgery-induced transient neuroinflammation caused long-lasting cognitive decline, and our findings are consistent with previous studies showing that spatial memory was impaired mainly from postoperative days 7–14 and restored on postoperative day 21 [35, 63]. In addition, we observed that the spatial memory deficit was generally restored to the control level on postoperative day 60, but some individual rats in the surgery group still exhibited impaired spatial recognition memory, suggesting that our experimental animal model mimicked core features of PNDs in humans, in which cognitive dysfunction is quite common in the first weeks following surgery but persists only in approximately 10% of elderly patients [64, 65]. Furthermore, spatial memory that strongly depends on the dHPC appeared to be more vulnerable than nonspatial memory to neuroinflammation in the present study, consistent with previous results [19, 35].

Although an increasing number of studies have shown an association between neuroinflammation and the development of PNDs [6–10], the mechanism by which surgery-induced transient neuroinflammation causes long-lasting cognitive decline is still a question of broad interest. Consistent with the previously reported effects of both acute and chronic neuroinflammation on the downregulation and hypofunction of NMDARs [31–33], we observed that neuroinflammation selectively led to a persistent downregulation of synaptic NR2A and NR2B subunits in the dHPC without altering the expression of extrasynaptic NMDARs. In contrast, we previously reported that a single intracerebroventricular injection of LPS did not alter NMDAR levels in the dHPC of adult rats [19]. Furthermore, another research group showed that neuroinflammation occurring after surgery upregulated NR2A and NR2B in total protein homogenates of the hippocampus from elderly mice [29, 66]. The possible explanations for these discrepancies are the use of animals with different ages and species, surgical techniques and ways to induce neuroinflammation (direct central administration of LPS or surgery-induced systemic inflammation). In addition to proinflammatory cytokines, many other neuromodulators may contribute to NMDARs changes after abdominal surgery as well. For example, reactive oxygen species (ROS), a mediator downregulating the expression of NMDARs [34], has been shown to be upregulated in the hippocampus of aged rats after laparotomy [67] and correlated with cognitive impairment after surgery [68]. Astrocyte, another important glial cell, can directly activate NMDARs by secreting the NMDAR ligands glutamate and D-serine after activation under neuroinflammation condition [69], it can also affect the NMDAR function via regulating the surface expression of NR2A and NR2B subunits [70].

Furthermore, in the present study, we observed dynamic changes in NMDAR subunits following surgery and found that the downregulation of NR2A and NR2B occurred concurrently with transient neuroinflammation induced by surgery and was temporally associated with spatial memory deficits postoperatively. However, the exact mechanism by which the synaptic NR2A and NR2B subunits were downregulated by transient neuroinflammation in our study is still unknown. Prolonged reduction in NMDAR was thought to be causally related to neuronal loss in various neurological diseases, such as traumatic brain injury, cerebral ischemia, and Alzheimer’s disease [71–73]. However, we observed that the downregulation of synaptic NR2A and NR2B did not result from neuronal loss on postoperative day 13, which was consistent with the results reported in the model of sepsis [74]. Hence, we speculated that other mechanisms may be involved in the persistent downregulation of NMDAR induced by transient neuroinflammation. Downstream molecules such as p38 and cAMP response element-binding protein (CREB) resulting from cytokine activation in the hippocampus are considered to be responsible for the long-lasting changes in NMDAR that are associated with NMDAR-dependent synaptic plasticity impairment and cognitive deficits [75]. Another possible interpretation is that NMDARs are in a high-risk state of change following neuroinflammation, because excessive glutamate is released from activated astrocytes [76]. These elevated glutamate levels may cause overstimulation of Ca^2+^ entry through NMDARs, subsequently downregulating NMDAR expression due to excitotoxicity [77]. Similar results in the model of closed head injury supported this hypothesis. An early upregulation of NMDAR was observed at 15 min after head injury and was short-lived, while 60 min after injury, progressive decreases in activated NMDAR were detected. This rapid shift of NMDAR levels may constitute the first lines of defense responded by the brain against the excessive stimulation of NMDAR after injury [78].

Typically, synaptic NMDARs (NR1/NR2A diheteromeric or NR1/NR2A/NR2B triheteromeric) are predominantly localized in the PSD, an electron-dense structure that is an important component of the dendritic spine head in excitatory synapses [79]. Functionally, synaptic NMDAR activation during learning stimulates downstream pathways. One of the best-characterized pathways involves the Rho family of small GTPases, including Rac1, Cdc42, and RhoA [80]. Rho GTPases act as synaptic molecular switches that transduce signals from presynaptic stimuli to the actin cytoskeleton. They exist in two states: a GTP-bound active state and a GDP-bound inactive state [56]. Activated Rho GTPases exert their regulator effects on actin cytoskeleton dynamics by binding and activating their downstream effectors, including Cofilin and Arp2/3 [57]. The actin cytoskeleton is the principal architectural component of the dendritic spine [81]. Dynamic remodeling of the actin cytoskeleton within dendritic spines is essential for activity-dependent structural changes in spines, termed structural plasticity, which is accepted to be the basis of learning and memory [82, 83]. Here, we focused on the structural plasticity of dendritic spines in the CA1 subregion of the dHPC following behavioral training, because Schaffer collateral-CA1 pyramidal cell synapses LTP is NMDAR-dependent [52], and the critical mediation of changes in the synaptic strength of these connections is likely derived from changes in the postsynaptic structure. We observed that surgery-induced neuroinflammation impaired the structural plasticity of dendritic spines and decreased the activity of the Rac1 signaling pathway during learning without altering the basal dendritic structure. We chose 40 min after behavioral training to study dendritic structural plasticity based on previous data showing prominent changes in apical dendritic spine density and morphology in normal rats at this timepoint [84]. In addition, Rac1, Cdc42, and RhoA are all activated by Ca^2+^/calmodulin-dependent protein kinase (CaMKII), an important downstream pathway of NMDAR activation in the early phase of LTP [59]. Nevertheless, only reduced Rac1 activity was observed in the dHPC following behavioral training in our present study. Different small GTPases may show different time courses of altered activity during structural changes in dendritic spine. Rac1 activation can persist for a longer time than Cdc42 and RhoA activation [59].

**Fig. 7:**
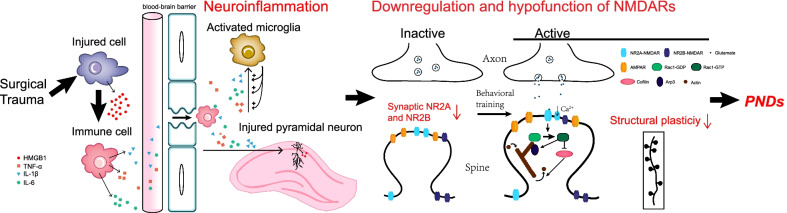
Proposed molecular mechanism underlying PNDs. Surgical trauma induces transient neuroinflammation by triggering the systemic inflammatory response. Then neuroinflammation causes long-lasting downregulation of synaptic NR2A and NR2B subunits in the dorsal hippocampus that is associated with synaptic NR2A and NR2B dysfunction, which is manifested by impaired structural plasticity of dendritic spine and decreased activated Rac1 (Rac1–GTP), phospho-cofilin, and Arp3 protein expressions. Ultimately, the persistent hypofunction of NR2A and NR2B leads to the development of long-lasting PNDs

The notable findings of the present study are that surgery-induced transient neuroinflammation causes persistent downregulation of synaptic NR2A and NR2B subunits associated with synaptic NMDAR dysfunction, subsequently leading to long-term spatial memory impairments through effects on Rac1 signaling pathways (Fig. 7). The contribution of neuroinflammation to the pathogenesis of PNDs was further supported by our pharmacological experiments, in which a specific HMGB1 inhibitor that directly inhibits the biological effect of active HMGB1 [60] reduced the systemic inflammatory response and dorsal hippocampal neuroinflammation, reversed synaptic NR2A and NR2B downregulation, and rescued spatial memory deficits. We found that anesthesia alone did not cause long-term cognition deficits, which was consistent with previous results [10, 35, 85]. Accumulating clinical evidences also showed that PNDs could occur irrespective of the anesthetic technique used, suggesting that surgery may play a prominent role in the development of postoperative cognitive deficits [86–88]. However, some studies reported that sevoflurane alone could induce cognitive decline in aged animals [89, 90]. Possible reason for this discrepancy is the different duration of general anesthetics used. The animals that exposed to 2% sevoflurane for 5–6 h exhibited impaired cognitive function [89, 90]. While the use of general anesthetics no more than 1 h neither induce peripheral or centrally proinflammatory cytokine or NMDARs changes [10, 35, 63, 85, 91], nor lead to postoperative cognitive dysfunction [10, 35, 63, 85]. Similarly, we combined use of sevoflurane and fentanyl to maintain anesthesia for about 40 min and found that anesthesia alone did not cause postoperative cognitive dysfunction.

## Conclusions

In summary, our results provide molecular, functional, behavioral, and pharmacological evidence that the downregulation and hypofunction of synaptic NMDARs, especially NR2A and NR2B subunits, induced by neuroinflammation after surgery are likely responsible for the development of PNDs in elderly animals. Clarification of the exact molecular mechanism underlying the transient neuroinflammation-induced long-lasting cognitive decline after surgery will tremendously provide effective treatment for PNDs.

## Supplementary Information

**Additional file 1:** **Table S1.** Total time of exploration in sample and test sessions in the OLM and ORM tests. **Fig. S1.** Spontaneous locomotor activity in the open field test following surgery. **Fig. S2. **Effect of surgical procedure without superior mesenteric artery clamping on spatial recognition memory. **Fig. S3.** Total time of object exploration and swimming speed in the behavioral test. **Fig. S4.** Expression of other NMDAR, AMPAR subunits, and synaptic proteins in the dHPC, the expression of NMDARs in other brain regions, and the number of pyramidal neurons and mature neurons in the dHPC. **Fig. S5.** Activity of other Rho GTPases in the dHPC during learning.

## Data Availability

The data sets used and/or analyzed are available from the corresponding author on reasonable request.
