# Supplementary material for: Transient neuroinflammation following surgery contributes to long-lasting cognitive decline in elderly rats via dysfunction of synaptic NMDA receptor
Source: J Neuroinflammation. 2022 Jul 13;19:181. doi: 10.1186/s12974-022-02528-5 (PMC9281167; doi:10.1186/s12974-022-02528-5)
Supplement: Supplementary file 1 — Additional file 1: Table S1. Total time of exploration in sample and test sessions in the OLM and ORM tests. Fig. S1. Spontaneous locomotor activity in the open field test following surgery. Fig. S2. Effect of surgical procedure without superior mesenteric artery clamping on spatial recognition memory. Fig. S3. Total time of object exploration and swimming speed in the behavioral test. Fig. S4. Expression of other NMDAR, AMPAR subunits, and synaptic proteins in the dHPC, the expression of NMDARs in other brain regions, and the number of pyramidal neurons and mature neurons in the dHPC. Fig. S5. Activity of other Rho GTPases in the dHPC during learning. [file 12974_2022_2528_MOESM1_ESM.pdf]

Supplementary Information

Title: **Transient neuroinflammation following surgery contributes to long-lasting cognitive decline in elderly rats via dysfunction of synaptic NMDA receptor**

Journal: Journal of Neuroinflammation

Authors: Bo Chen, Guangcheng Qin, Jingyu Xiao, Xiaoyuan Deng, Aolei Lin, Hongliang Liu

Correspondence to: Hongliang Liu, PhD., Department of Anesthesiology, Chongqing University Cancer Hospital, Chongqing, 400030, P.R. China.

E-mail: light3748@sina.com

Supplementary Table 1. Total time of exploration in sample and test sessions in the OLM and ORM tests.

Time	Group	OLM		ORM	
		1 h delay		1 h delay	
		S (s)	T (s)	S (s)	T (s)
7 d	Control	49.1±3.8	20.7±1.9	51.0±4.4	20.5±2.2
	Surgery	51.7±3.1	23.5±2.3	50.0±2.9	20.7±2.1
14 d	Control	42.9±5.8	17.4±3.2	46.1±6.6	14.8±2.6
	Surgery	49.8±4.4	17.4±3.7	50.3±5.0	14.8±3.0
21 d	Control	45.9±8.4	24.5±3.4	46.3±7.9	19.7±2.2
	Surgery	44.3±5.5	23.0±2.1	47.4±6.0	20.1±2.4
60 d	Control	49.3±6.9	22.1±3.6	47.0±7.3	25.4±2.6
	Surgery	54.3±8.7	22.7±3.5	47.4±6.8	21.0±1.9

OLM, object location memory, ORM, object recognition memory, S, sample session, T, test session. Data are presented as means±SEM and statistical analyzed by a two-way ANOVA followed by a Tukey's test.

Supplementary Figures

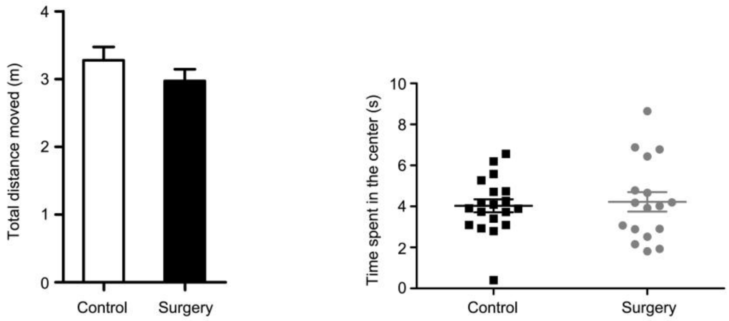

Supplementary Fig. 1. The spontaneous locomotor activity in the open field test following surgery. (a) Total distance moved and (b) total time spent in the center in the open field test on postoperative Day 14 (Control, n=19, Surgery, n=17). Data are presented as means±SEM and statistical analyzed by a Student's t test.

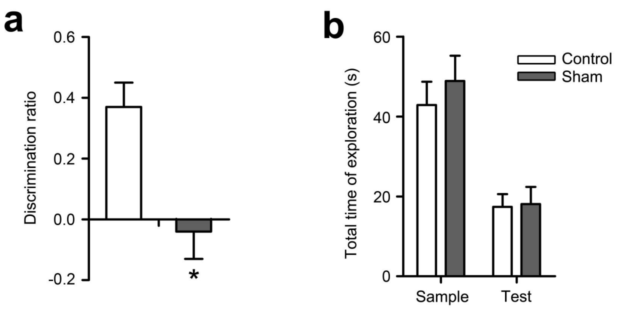

Supplementary Fig. 2. The effect of surgical procedure without superior mesenteric artery clamping on spatial recognition memory. (a) The DR in the OLM on postoperative Day 14. (b) Total time of object exploration in the sample and test sessions of the OLM test on postoperative Day 14 (Control, n=9, Sham, n=9). Data are presented as means±SEM and statistical analyzed by a Student's t test. *p<0.05 versus Control.

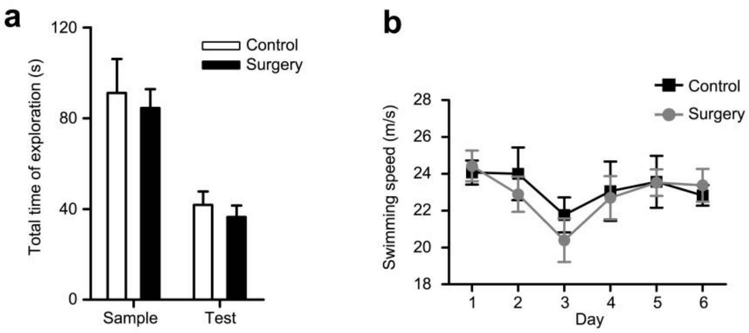

Supplementary Fig. 3. Total time of object exploration and swimming speed in the behavioral tests. (a) Total time of object exploration in the sample and test sessions of the OIP test on postoperative Day 14. (b) Swimming speed in the MWM from postoperative Days 9 to 14. Data are presented as means±SEM and statistical analyzed by a Student's t test (a) or a repeated ANOVA followed by a Tukey's test (b).

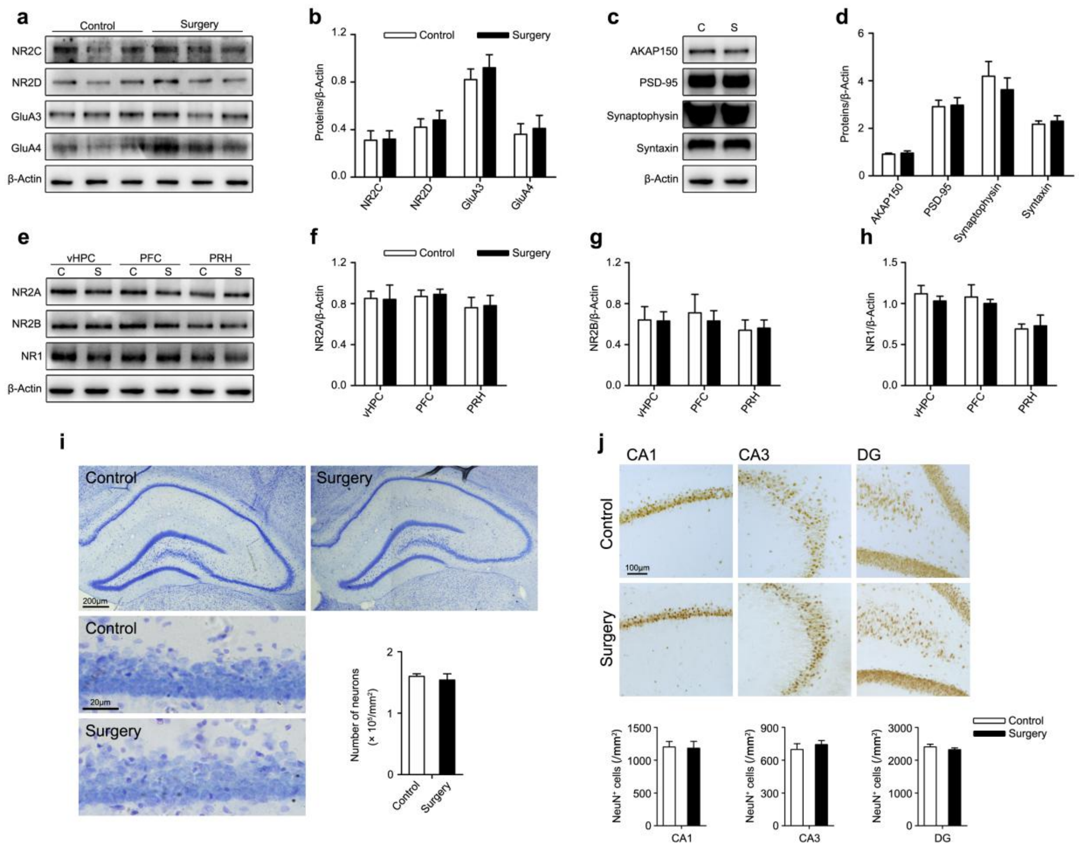

Supplementary Fig. 4. The expression of other NMDAR, AMPAR subunits, and synaptic proteins in the dHPC, the expression of NMDARs in other brain regions, and the number of pyramidal neurons and mature neurons in the dHPC. (a,b) Representative immunoblots and corresponding densitometric analysis of NR2C, NR2D, GluA3, and GluR4 proteins in the dHPC on postoperative Day 13 (n=6 per group). (c,d) Representative immunoblots and corresponding densitometric analysis of AKAP150, PSD-95, synaptophysin, and syntaxin proteins in the dHPC on postoperative Day 13 (n=6 per group). (e-h) Representative immunoblots and corresponding densitometric analysis of NR2A, NR2B, and NR1 proteins in the vHPC, PFC, and PRH on postoperative Day 13 (n=6 per group). (i) Representative 10× and 40× objectives images of nissl staining and quantitative analysis of pyramidal neurons in the dHPC on postoperative Day 13 (n=6 per group). (j) Representative 20× objective images of immunohistochemistry and quantitative analysis of NeuN^{+} cells (a mature neuron marker) in the CA1, CA3, and DG of dHPC on postoperative Day 13 (n=6 per group). C control,

S surgery, vHPC ventral hippocampus, PFC prefrontal cortex, PRH perirhinal cortex. Data are presented as means ± SEM and statistical analyzed by a Student's t test (b, d, f, g, h, i, and j).

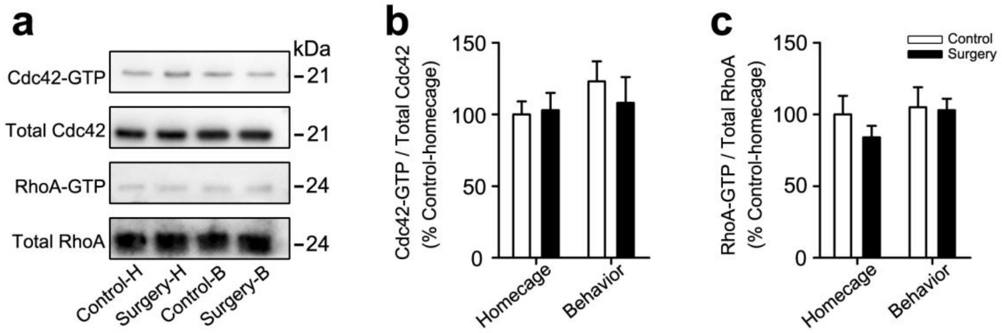

Supplementary Fig. 5. The activity of other Rho GTPases in the dHPC during learning. (a-c) Representative immunoblots and corresponding densitometric analysis of Cdc42-GTP, total Cdc42, RhoA-GTP, and total RhoA proteins in the dHPC during learning on postoperative Day 14 (n=6 per group). Control-H control-homecage, Surgery-H surgery-homecage, Control-B control-behavior, Surgery-B surgery-behavior. Data are presented as means ± SEM and statistical analyzed by a one-way ANOVA followed by a Tukey's test (b and c).
